# LC‐Pred: A Transformer‐Based Interactive Interface for Liver Cirrhosis Prediction

**DOI:** 10.1155/ijh/3655128

**Published:** 2026-05-23

**Authors:** Bisweswari Rath, Satya Ranjan Dash, Rajani Kanta Mahapatra

**Affiliations:** ^1^ School of Biotechnology, KIIT Deemed To Be University, Bhubaneswar, India, kiit.ac.in; ^2^ School of Computer Application, KIIT Deemed To Be University, Bhubaneswar, India, kiit.ac.in

**Keywords:** AI, Child–Pugh, clinical data, hepatology, LC, liver cirrhosis, MELD, noninvasive diagnosis, transformer model, web application

## Abstract

**Objectives:**

Incorporating transformer, an innovative deep learning–based model with an authenticated and user‐friendly client‐server web application namely LC‐Pred, this study highlights the early prediction of liver cirrhosis (LC), which will be beneficial for both the clinicians and the LC patients. LC‐Pred delivers both single and bulk prediction abilities making it fast, sturdy, and secure to be used in healthcare sectors.

**Methods:**

This study uses a total of 1098 real‐time patients′ data having both LC and nonliver cirrhosis (NLC) cases to implement and compare traditional scoring systems and AI‐based models, by using 20 clinical parameters with two demographic data such as patients′ age and gender.

**Results:**

The tool consists of authentication, PDF report generation and spontaneous interface elevated for clinical workflow incorporation. Transformer model exhibits the highest accuracy among all the traditional and artificial intelligence (AI) models and is selected to be linked with LC‐Pred for classification of cirrhosis. Transformer model accomplishes a vigorous performance with precision recall area under the curve (PR‐AUC) of 0.907, receiver operating characteristics area under the curve (ROC‐AUC) of 0.989, sensitivity/recall of 0.857 (for LC detection) and specificity of 0.947 (for NLC prediction), Brier score is of 0.027 and test accuracy of 0.977.

**Conclusion:**

The tool exhibits noteworthy upgradation in AI‐assisted hepatology, over traditional scoring techniques like model for end‐stage liver disease (MELD) and Child–Pugh, by stabilizing the technical intricacy with clinical efficacy. This note delineates the application framework, model training principles, evaluating results and the importance of implementing an aligned AI system to be utilized by the clinicians.

## 1. Introduction

Being a crucial health burden worldwide, early diagnosis and treatment of liver cirrhosis (LC) is clinically challenging. As a blood‐borne illness, and being contaminating through people′s blood or blood‐containing body fluid, globally around 71 million individuals are infected with chronic hepatitis C, which is the leading cause of LC and 399,000 deaths happening every year [1]. Long‐term liver damage and excess intake of alcohol give rise to LC. Heavy intake of alcohol is basically associated with the development of LC [2, 3]. Being a chronic and difficult‐to‐reverse conditioned disease, LC progresses over time [4–7]. Although cirrhosis may remain clinically silent in its compensated stage, its progression results in complications such as ascites, jaundice, gastrointestinal bleeding, hepatic encephalopathy, and a high risk of hepatocellular carcinoma (HCC) [8, 9]. Furthermore, geographical disparity being the key factor of increased mortality, innovative solutions have to be made for the people living in more rural and low‐income areas.

Traditional diagnostic methods for LC detection such as intrusive liver biopsy, transient elastography (FibroScan), and scoring systems like Child–Pugh score and MELD are being used. Noninvasive diagnostic methods like magnetic resonance elastography (MRE) offer high accuracy in detecting the severity of LC; however, it is challenging for adoption as a consequence of its longer evaluation window, advanced equipment installment, and limited accessibility in primary healthcare [10–13]. Hence, in this digital era, early detection of LC using artificial intelligence (AI) in clinical datasets [14, 15] can avert the progression of the disease and mortality.

Notwithstanding the advanced algorithms, a constant disparity exists between well‐performed model (in research) and its need in healthcare sectors, because of their inaccessibility in the real‐time world. To bridge this gap, this project has focused on the integrated, robust, secure, and deployable AI system LC‐Pred, which is the ultimate need as a clinical support system. This system is designed based on the needful requirements like smooth and simple usage with clear results, efficient batch processing, safety, and security. The following are the detailed idea about how we functionalized an AI‐based web tool, which will be helpful for clinicians in predicting LC at the earliest.

## 2. Data, Software, and Model Implementation

### 2.1. Data Collection and Preprocessing

This single‐institutional retrospective study conformant with the Health Insurance Portability and Accountability Act (HIPAA), which regulates the safety regarding the usage of patients′ information for research and analysis, was approved by the institution′s ethical committee, assuring that all the guidelines comply with the established ethical constraints. Patients′ data, with both confirmed diagnosis of LC and nonliver cirrhosis (NLC), were collected from the Kalinga Institute of Medical Sciences (KIMS), Bhubaneswar, Odisha with a duration of 42 months starting from January 2022 to December 2025 and stored in the Structured Query Language (SQL)–based database through our custom built electronic health record (EHR) with proper security and authentication. Data collection was exclusively done from the Department of Gastroenterology and Hepatology and the Medical Record Department (MRD) of KIMS, from which a total of 992 LC and 106 NLC patients with the most relevant data (having required laboratory parameters) were extracted for our model′s inclusion criteria, maintaining the accuracy and pertinence of our findings. Proceeding with the consultations provided by the medical professionals, we determined the scope of relevant data by obtaining precise demographic data such as age and gender in conjunction with the hematological and biochemical parameters. The hematological parameters include RDW‐SD, RDW‐CV, HbA1c, FBS, PPBS, serum hemoglobin, INR and platelet count whereas biochemical parameters consist of serum albumin, total serum bilirubin, direct serum bilirubin, serum urea, serum creatinine, serum total protein, serum sodium, serum potassium, ALT (also known as SGPT), AST (also called as SGOT), GGT, and ALP. These 22 clinical and biomarkers data are finalized for their influential role in analyzing the liver function and inflammation, under the physician′s supervision. This targeted strategy validated that our dataset is both extensive and germane to the milestone of our study, enhancing rigorous analysis while advancing the LC assessment.

### 2.2. Comparison Among Traditional Methods and AI Models—Final Model Selection

Mortality rate for critically ill cirrhotic patients in the intensive care units (ICUs), due to liver dysfunction and multiple organ failure, can reach up to 45%, with some cases exceeding 70%; hence it has been a remarkable challenge for the physicians to take intensive actions to treat the critically ill cirrhotic patients with complications and comorbidities and can be effectively predicted by robust and interpretable web‐based AI models [16–21]. AI tools intensely showcase their competence to assess laboratory parameters and patients′ data. Their ability to substantially improve diagnostic processes has earned notable consideration from healthcare providers and researchers. Hence, to get better prediction performance, a comparison has been made between traditional models and AI models. Traditional scoring systems include various versions of MELD and Child–Pugh, whereas AI models consist of logistic regression (LR), random forest (RF), extreme gradient boosting (XGBoost), GradientBoost, and transformer. MELD is a prediction‐based traditional scoring system that uses bilirubin, creatinine, and INR to estimate the short‐duration mortality. In MELD‐Na, the further version of MELD, sodium is added to the existing parameters of MELD for better estimation. MELD 3.0, the latest version of MELD, uses six parameters by adding two more parameters, namely sex and albumin, to MELD‐Na, increasing efficacy in cirrhosis detection. Child–Pugh score, another traditional scoring system, is computed by combining five parameters such as bilirubin, creatinine, INR, ascites, and hepatic encephalopathy to determine the severity of the liver disease. However, due to the absence of ascites and hepatic encephalopathy parameters in our dataset, we computed modified Child–Pugh score by taking bilirubin, creatinine, and INR. LR shows better performance in binary classification and uses L1/L2 regularization, whereas RF is the integration of multiple decision trees, and both of them prevent overfitting. GradientBoosting classifier builds decision trees in a staged manner while correcting the errors of previous ones, whereas XGBoost is the optimized version of the GradientBoost with embedded regularization and productive processing power. Being a pretrained deep learning model, the transformer model uses a self‐attention mechanism, which works well with unstructured and imbalanced data and has shown the highest accuracy in comparison to the ML models with a lower marginal difference, whereas traditional methods fail to achieve considerable performance due to less feature selection and lack of ability in handling high data imbalance like AI models. Thus, we incorporated our prediction tool with the transformer model which effectively handles the imbalanced and real‐time medical data with enhanced accuracy.

### 2.3. Overview of System Architecture

The system is based on a client‐server model consisting of the following:

Backend/server: Python Flask is used which provides Representational State Transfer application programming interface (RESTful API) endpoints for authentication, prediction of LC, showing results and report creations.

Frontend/client: dynamic hypertext markup language (HTML), Cascading Style Sheets (CSS), and JavaScript (JS) puts forth Single Prediction, Bulk Upload, Results, and About.

AI model: Transformer model fitted with scaler, imputational and label encoders, wrapped in a single interface for further LC prediction.

Data pipeline: For cleaning and preprocessing of raw patient data to match with the model′s expected input schema.

### 2.4. Usage of Web Tool

#### 2.4.1. Backend Formation

For clean segregation of operationality of the web tool, Flask is used. Authentication mechanism offers Login and Logout features to be securely used by the clinicians. “Single Prediction” accepts and returns JavaScript Object Notation (JSON) formatted data and makes prediction using ModelWrapper. “Bulk prediction” feature works on multiple patient data and does batch processing for predicting the large dataset at once using ModelWrapper. It accepts both comma‐separated values (CSV) and Excel files of patients data and an Excel file is being downloaded to the system, after prediction, with two added new columns for prediction, which has values LC/NLC and Probability_LC(%), which ranges from 0 to 100 based on the prediction probability, higher for LC cases and lower for NLC cases. Postprocessing with openpyxl is an essential factor which applies a light orange highlighting in all the NLC rows improvising visualization for the users. Report generation, consisting of a header, accepts JSON formatted results and patients metadata and creates a downloadable professional report in portable document format (PDF) format, which includes a heading, a structured table of lab test values, summary of patient, and recent predictions. “__init__.py”, being an entry point, properly handles the Flask app.

#### 2.4.2. Frontend Creation

**Figure 1:**
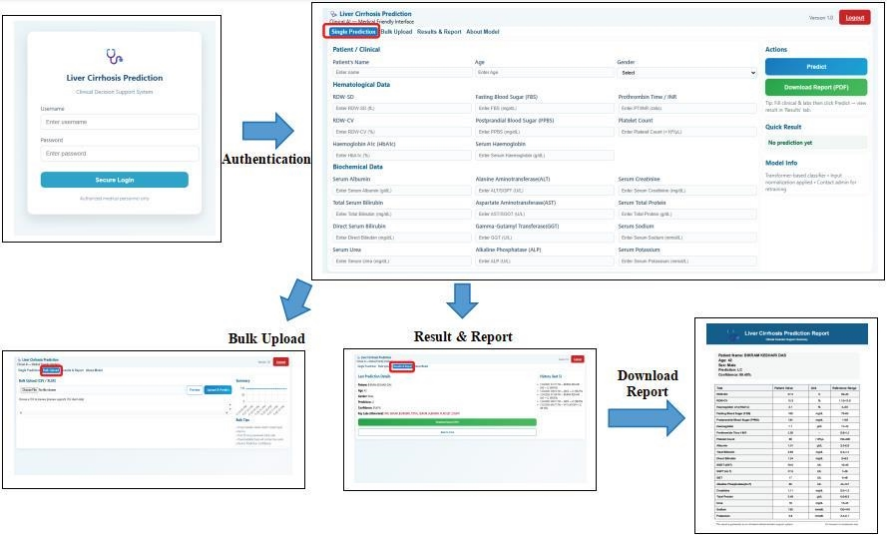
Overview of LC‐Pred and its main features. Top: left window represents authentication options. Top: main window demonstrates single prediction features and other options. Bottom left shows bulk upload options. Bottom center displays the result and report download options. Bottom right presents the downloaded lab report of patient.

The web tool, LC‐Pred (shown in Figure 1), is created for the ease of access to the model by the clinicians without any confusion in the complexity of the model.

As presented in Figure S1, the single prediction page is organized as two panels consisting of form card and side bars. Three‐column grid sections of the form card include Clinical Data, Hematological Data, and Biochemical Data. Sidebar has the primary action features like Predict button, Download Report, Quick Result, and model′s information. Predict button is responsible for sending all the inputted data via AJAX and displays the result (LC/NLC) in “Quick Result” field, which further can be downloaded in PDF format by clicking the “Download Report” button.

In Bulk Prediction section “Choose File” field, for uploading the files, accepts data in both the CSV and Excel format. Preview Button offers the visual functionality of the uploaded data file in a table format where as “Upload & Predict” button is responsible for downloading the Excel formatted file of all the patients′ data with two new extra columns, Prediction and Probablility_LC (%). Sidebar has the summary visualization feature that shows the graph of prediction confidence(%) with the tips of bulk prediction which will ease the understanding of the end users. The overview of the bulk prediction option is showcased in Figure S2.

Results and Report section presents a simple and more readable visualization format of patient details with Report Downloading in PDF format, depicted in the Figure S3, and Back to Form action buttons with history of last five predictions, demonstrated in Figure S4.

#### 2.4.3. Transformer Model Implementation Using Clinical Data

Using PyTorch, transformer—a deep learning–based model is implemented, which classifies LC as Class 0 and NLC as Class 1, as computer only understands 0/1 format. It uses Attention mechanism, which learns the most complex patterns among input features for accurate prediction. By building a more rigorous diagnostic model from the clinical lab test values, AI has proven its potential ability to break the linkage between accuracy and cost that offers an adaptable solution to the current screening gap [22].

Unlike traditional models, this model consists of multihead self‐attention (MHSA), feed‐forward network (FFN), residual connection, and layer normalization. The model uses four self‐dependent heads in MHSA, enabling it to learn relationships among the components of the input sequences. FFN, a fully connected neural network, processes and transforms each feature after attention, enhancing nonlinearity. Residual connections help in preserving the important information, hence showcasing better performance and ensuring smooth gradient flow at the time of back‐propagation. Unlike the batch normalization, layer normalization is independent of the batch size and is applied after each residual connection to sustain and accelerate the training. The encoder features are bundled up to form a patient‐level representation.

The output layer produces logit score, continuous values (−∞ to +∞), which are then passed through the sigmoid function to be classified as 0/1 format. Transformer model is trained and validated on the static dataset of 1098 patients, which consists of 992 LC data and 106 NLC data. This huge data imbalance is avoided by applying the BCEWithLogitsLoss function that applies both the Sigmoid and binary cross entropy loss (BCEloss) to prevent the model′s misclassifications. As the dataset is highly imbalanced, the prediction will be more prone to classifying the majority class, that is, LC. To avoid this wrong prediction, we have passed *Pos_weight* (shown below) in the BCEWithLogitsLoss function that will boost the prediction of the minority class, ameliorating sensitivity and ROC‐AUC.

Pos_weight= number of LC casesnumber of NLC cases

Adam Optimizer with 1 × 10^−3^ (0.001) learning rate showcases that Adam is changing the model′s weight by smaller steps of 0.001 throughout the training. A scheduler named ReduceLROnPlateau is used to lessen the learning rate or stop training the model when the improvement of the model is too slow or stops accordingly, hence it prevents overfitting. A smaller learning rate is typically used for finding the best solution as it learns very deeply. The overfitting of the model is notably abstained by monitoring validation loss in each epoch, early stopping if improvement of the model stops, and saving the best weight. To build the model, 80% of the dataset is taken for training and 20% for testing purposes. ROC‐AUC and accuracy on the set have proven the model′s remarkable performance. Label encoding, median imputation, and standard scaling are used for categorical features, handling missing values and normalization, respectively.

## 3. Results

The whole system is relied on the performance of the transformer model which has been calculated on the basis of evaluation metrics such as accuracy, PR‐AUC (precision recall area under the curve), ROC‐AUC (receiver operating characteristics area under the curve), sensitivity/recall, specificity, precision, F1‐score, Brier score, and confusion matrix.

As per Table 1, several traditional methods and AI models were compared from which transformer model has shown better diagnostic ability in detecting LC and NLC. Clinical scoring systems were unable to classify mortality cases, resulting in lower scores in overall performance. All the models were trained on 80% of the dataset and validated on the rest 20% of data for models′ evaluation. Though the compared AI‐models illustrated equivalent discriminative ability with only marginal variations, transformer model attained the highest predictive ability, by securing accuracy of 0.977, PR‐AUC of 0.907, and ROC‐AUC of 0.989, among all. The least brier score of 0.027, among all the compared models, highlights that the model is properly calibrated and the predicted probabilities are quite accurate for real‐world patients′ data.

**Table 1:** Comparison among evaluation metrics of several traditional scoring methods and AI models.

Metric	Transformer model	Logistic regression	Random forest	Gradient boost	XGBoost	MELD	MELD‐Na	MELD 3.0	Child–Pugh
**Accuracy**	0.977	0.953	0.942	0.907	0.965	0.593	0.616	0.581	0.709
**ROC-AUC**	0.989	0.964	0.952	0.980	0.951	0.788	0.799	0.709	0.775
**PR-AUC**	0.907	0.839	0.845	0.778	0.836	0.497	0.478	0.379	0.502
**Sensitivity/recall**	0.857	0.714	0.571	0.286	0.857	0.619	0.619	0.524	0.571
**Specificity**	0.947	0.987	0.993	0.993	0.980	0.589	0.616	0.589	0.728
**Precision**	0.857	0.882	0.923	0.857	0.857	0.173	0.183	0.151	0.226
**F1 score**	0.900	0.789	0.706	0.429	0.857	0.271	0.283	0.234	0.324
**Brier score**	0.027	0.045	0.047	0.056	0.124	0.446	0.339	0.530	0.315

Specificity, also known as true negative rate (TNR), means from all the NLC predictions how many are correctly predicted as NLC by the model, and sensitivity, also known as true positive rate (TPR), calculates the model′s accuracy rate of identifying the positive data (here LC). Transformer model has obtained the specificity of 0.947 and sensitivity of 0.857, based on the test set, which demonstrates the model′s vigorous distinguishing ability between LC and NLC cases.

According to Figure S5, confusion matrix of transformer model, true positive (TP) and true negative (TN) are correctly identified as LC and NLC, respectively, and false positives (FP) and false negative (FN) are minimized by applying the proper threshold, computed by the model.

Both in single and bulk predictions, from submitting the data to getting the results takes a couple of milliseconds, making the LC‐Pred tool more robust, interactive, and efficient to be used in healthcare sectors.

## 4. Discussion

To bridge the significant quality gaps in chronic LC management, an attractive and potentially scalable prospective intervention could help standardize clinical care in hepatology [23]. Thus, the study encapsulates the transformer model into a client‐server tool which will be used by hepatologist (liver specialists), gastrologists (who treat the whole digestive system), and also other clinicians without any basic technical knowledge. Bulk prediction feature will enhance workflow in the hospital sectors as it allows hundreds of patient records simultaneously reducing the time consumption. A single prediction report (visualized in Figure S4) contains patient name, age, gender, prediction and model′s confidence with all the laboratory test data having their corresponding inputted values, units, and reference ranges, making the report convenient and understandable.

### 4.1. Limitations and Future Work

Selected model is trained on static data. To improvise the disease′s progression over time, longitudinal data would be the key factor to be considered. This model is trained on single‐centered data. However, subsequent analysis of this research aims for focusing on the real time data from different demographic areas to strengthen the diversity and generalization capability of the data. Our study is based on only clinical laboratory test values. Medical images like magnetic resonance imaging (MRI) or CT scan images will be included in our further study to make the model robust and vigorous. To obtain generalizability, we searched for some independent datasets. Yet, we recognized the most similar dataset that includes only eight out of the 22 parameters utilized in our models. Due to the unavailability of an identical set of predictors in the public cohort, performing external validation, to avoid unbiased estimations of the model, is still a limitation in our research.

In the web tool, the history of patients is stored as JSON in a temporary local file. However, the database can be connected to store the patient′s information over their every visit to keep track of the intensity of the disease. As a stand‐alone application, it would be connected to the EHRs of the healthcare sectors to derive the data easily for cirrhosis prediction. However, the varying real‐time data of the patients will need preprocessing before the prediction, as the test reports of different hospitals and clinics would vary, which will be more time‐consuming and expensive.

## 5. Conclusion

The system′s evaluation metrics exhibit potential distinction between LC and NLC that adequately analogizes with both the conventional scoring systems and the alternative AI technique. Existence of some limitations still signifies the distinct track in the subsequent improvement. This study illustrates that being integrated with an interactive interface, transformer model, an advanced deep learning framework, is a real transformation of AI from conceptual analysis to empirical benefit in the healthcare sectors for accurate and in‐time treatment of LC while reducing the bridge in between real‐time and accurate prognosis of the disease and preserving the smooth adoption for the clinicians without any proper technical expertise.

**Nomenclature**
AI: artificial intelligence
ALP: alkaline phosphatase
ALT: alanine aminotransferase
AST: aspartate aminotransferase
BCEloss: binary cross entropy loss
CSS: Cascading Style Sheets
CSV: comma‐separated values
CT: computed tomography
EHR: electronic health record
FBS: fasting blood sugar
FFN: feed‐forward network
FP: false positive
FN: false negative
GGT: gamma‐glutamyl transferase
HbA1c: glycated hemoglobin
HCC: hepatocellular carcinoma
HIPAA: Health Insurance Portability and Accountability Act
HTML: hypertext markup language
ICU: intensive care unit
INR: international normalized ratio
JS: JavaScript
JSON: JavaScript Object Notation
KIMS: Kalinga Institute of Medical Sciences
LC: liver cirrhosis
LR: logistic regression
MELD: model for end‐stage liver disease
MELD‐Na: model for end‐stage liver disease—sodium
MHSA: multihead self‐attention
MPR: magnetic resonance imaging
MRD: medical record department
MRE: magnetic resonance elastography
NLC: nonliver cirrhosis
PDF: Portable Document Format
PPBS: Postprandial Blood Sugar
RDW‐CV: red cell distribution width—coefficient of variation
PR‐AUC: precision recall area under the curve
RDW‐SD: red cell distribution width—standard deviation
RESTful API: Representational State Transfer Application Programming Interface
RF: random forest
ROC‐AUC: receiver operating characteristics area under the curve
SGOT: serum glutamic oxaloacetic transaminase
SGPT: serum glutamic pyruvic transaminase
SQL: Structured Query Language
TE: transient elastography
TN: true negative
TP: true positive
TNR: true negative rate
TPR: true positive rate
XGBoost: extreme gradient boosting

## Funding

This study was supported by the Department of Science and Technology, Ministry of Science and Technology, India (10.13039/501100001409) (CRG/2022/006954).

## Ethics Statement

This study was approved by the Institutional Ethics Committee at the Kalinga Institute of Medical sciences (Ref No: KIIT/KIMS/IEC/1347/2023, approved date: 08/05/2023) with a waiver consent from the committee.

## Consent

Patient consent forms are submitted to the hospital.

## Conflicts of Interest

The authors declare no conflicts of interest.

## Supporting information


**Supporting Information** Additional supporting information can be found online in the Supporting Information section. 

## Data Availability

The data that support the findings of this study are available on request from the corresponding author. The data are not publicly available due to privacy or ethical restrictions.

## References

[1] Jefferies M. , Rauff B. , Rashid H. , Lam T. , and Rafiq S. , Update on Global Epidemiology of Viral Hepatitis and Preventive Strategies, World Journal of Clinical Cases. (2018) 6, no. 13, 589–599, 10.12998/wjcc.v6.i13.589, 2-s2.0-85057342392, 30430114. 30430114 PMC6232563

[2] Niu X. , Zhu L. , Xu Y. , Zhang M. , Hao Y. , Ma L. , Li Y. , and Xing H. , Global Prevalence, Incidence, and Outcomes of Alcohol Related Liver Diseases: A Systematic Review and Meta-Analysis, BMC Public Health. (2023) 23, no. 1, 10.1186/s12889-023-15749-x, 37170239. PMC10173666 37170239

[3] Llamosas-Falcon L. , Probst C. , Buckley C. , Jiang H. , Lasserre A. M. , Puka K. , Tran A. , Zhu Y. , and Rehm J. , How Does Alcohol Use Impact Morbidity and Mortality of Liver Cirrhosis? A Systematic Review and Dose–Response Meta-Analysis, Hepatology International. (2024) 18, no. 1, 216–224, 10.1007/s12072-023-10584-z, 37684424. 37684424 PMC10920389

[4] Tran A. , Jiang H. , Lange S. , Llamosas-Falcón L. , Petkevičienė J. , Radišauskas R. , Štelemėkas M. , and Rehm J. , How Does Taxation Affect Liver Cirrhosis Across Age Groups? An Analysis of Alcohol Control Policies on Liver Cirrhosis Outcomes in Lithuania Between 2001 and 2022, Alcohol and Alcoholism. (2025) 60, no. 4, 10.1093/alcalc/agaf034, 40561461. PMC12192443 40561461

[5] Lelbach W. K. , Cirrhosis in the Alcoholic and Its Relation to the Volume of Alcohol Abuse, Annals of the New York Academy of Sciences. (1975) 252, no. 1, 85–105, 10.1111/j.1749-6632.1975.tb19146.x, 2-s2.0-0016861504. 1096716

[6] Rehm J. , Patra J. , Brennan A. , Buckley C. , Greenfield T. K. , Kerr W. C. , Manthey J. , Purshouse R. C. , Rovira P. , Shuper P. A. , and Shield K. D. , The Role of Alcohol Use in the Aetiology and Progression of Liver Disease: A Narrative Review and a Quantification, Drug and Alcohol Review. (2021) 40, no. 7, 1377–1386, 10.1111/dar.13286, 33783063. 33783063 PMC9389623

[7] Dezső K. , Paku S. , Kóbori L. , Thorgeirsson S. S. , and Nagy P. , What Makes Cirrhosis Irreversible?—Consideration on Structural Changes, Frontiers in Medicine. (2022) 9, 876293, 10.3389/fmed.2022.876293, 35572980. 35572980 PMC9091510

[8] Raghavan R. P. , Alexander K. T. , Sadasivan S. , Parmar C. , and Kathirvel M. , Genetic Variants in Liver Cirrhosis: Classifications, Mechanisms, and Implications for Clinical Practice, Journal of Personalized Medicine. (2026) 16, no. 1, 10.3390/jpm16010029. PMC12842787 41590522

[9] Angeli P. , Bernardi M. , Villanueva C. , Francoz C. , Mookerjee R. P. , Trebicka J. , Krag A. , Laleman W. , and Gines P. , EASL Clinical Practice Guidelines for the Management of Patients With Decompensated Cirrhosis, Journal of Hepatology. (2018) 69, no. 2, 406–460, 10.1016/j.jhep.2018.03.024, 2-s2.0-85045100709, 29653741. 29653741

[10] Zhang H. , Hao E. , Xia D. , Ma M. , Wu J. , Liu T. , Gao M. , and Wu X. , Estimating Liver Cirrhosis Severity With Extracellular Volume Fraction by Spectral CT, Scientific Reports. (2025) 15, no. 1, 18343, 10.1038/s41598-025-03717-x, 40419616. 40419616 PMC12106838

[11] Ajmera V. H. , Liu A. , Singh S. , Yachoa G. , Ramey M. , Bhargava M. , Zamani A. , Lopez S. , Mangla N. , Bettencourt R. , Rizo E. , Valasek M. , Behling C. , Richards L. , Sirlin C. , and Loomba R. , Clinical Utility of an Increase in Magnetic Resonance Elastography in Predicting Fibrosis Progression in Nonalcoholic Fatty Liver Disease, Hepatology. (2020) 71, no. 3, 849–860, 10.1002/hep.30974, 31556124. 31556124 PMC7828573

[12] Kupczyk P. A. , Mesropyan N. , Isaak A. , Endler C. , Faron A. , Kuetting D. , Sprinkart A. M. , Mädler B. , Thomas D. , Attenberger U. I. , and Luetkens J. A. , Quantitative MRI of the Liver: Evaluation of Extracellular Volume Fraction and Other Quantitative Parameters in Comparison to MR Elastography for the Assessment of Hepatopathy, Magnetic Resonance Imaging. (2021) 77, 7–13, 10.1016/j.mri.2020.12.005, 33309923. 33309923

[13] Wang X. P. , Wang Y. , Ma H. , Wang H. , Yang D. W. , Zhao X. Y. , Jin E. H. , and Yang Z. H. , Assessment of Liver Fibrosis With Liver and Spleen Magnetic Resonance Elastography, Serum Markers in Chronic Liver Disease, Quantitative Imaging in Medicine and Surgery. (2020) 10, no. 6, 1208–1222, 10.21037/qims-19-849, 32550131. 32550131 PMC7276364

[14] Gananandan K. , Kazankov K. , Tapper E. B. , and Mookerjee R. P. , The New Digital Era in Decompensated Cirrhosis, Lancet Digital Health. (2025) 7, no. 1, e54–e63, 10.1016/S2589-7500(24)00174-2, 39572283. 39572283

[15] Nayak A. , Baidya Kayal E. , Arya M. , Culli J. , Krishan S. , Agarwal S. , and Mehndiratta A. , Computer-Aided Diagnosis of Cirrhosis and Hepatocellular Carcinoma Using Multi-Phase Abdomen CT, International Journal of Computer Assisted Radiology and Surgery. (2019) 14, no. 8, 1341–1352, 10.1007/s11548-019-01991-5, 2-s2.0-85065476271. 31062266

[16] Wang Z. , Li F. Y. , Cai J. , Xue Z. , Zhou Y. , and Wang Z. , Construction and Validation of a Machine Learning-Based Prediction Model for Short-Term Mortality in Critically Ill Patients With Liver Cirrhosis, Clinics and Research in Hepatology and Gastroenterology. (2025) 49, no. 1, 102507, 10.1016/j.clinre.2024.102507, 39622289. 39622289

[17] Wehler M. , Kokoska J. , Reulbach U. , Hahn E. G. , and Strauss R. , Short-Term Prognosis in Critically Ill Patients With Cirrhosis Assessed by Prognostic Scoring Systems, Hepatology. (2001) 34, no. 2, 255–261, 10.1053/jhep.2001.26522, 2-s2.0-0034922612, 11481609. 11481609

[18] Jenq C. C. , Tsai M. H. , Tian Y. C. , Lin C. Y. , Yang C. , Liu N. J. , Lien J. M. , Chen Y. C. , Fang J. T. , Chen P. C. , and Yang C. W. , RIFLE Classification Can Predict Short-Term Prognosis in Critically Ill Cirrhotic Patients, Intensive Care Medicine. (2007) 33, no. 11, 1921–1930, 10.1007/s00134-007-0760-6, 2-s2.0-35448952666, 17605129. 17605129

[19] Avadhanam M. and Kulkarni A. V. , Intensive Care Unit Care of a Patient With Cirrhosis, Medical Clinics. (2023) 107, no. 3, 567–587, 10.1016/j.mcna.2022.12.006, 37001954. 37001954

[20] Levesque E. , Hoti E. , Azoulay D. , Ichaï P. , Habouchi H. , Castaing D. , Samuel D. , and Saliba F. , Prospective Evaluation of the Prognostic Scores for Cirrhotic Patients Admitted to an Intensive Care Unit, Journal of Hepatology. (2012) 56, no. 1, 95–102, 10.1016/j.jhep.2011.06.024, 2-s2.0-83555162520, 21835136. 21835136

[21] Thomson S. J. , Moran C. , Cowan M. L. , Musa S. , Beale R. , Treacher D. , Hamilton M. , Grounds R. M. , and Rahman T. M. , Outcomes of Critically Ill Patients With Cirrhosis Admitted to Intensive Care: An Important Perspective From the Non-Transplant Setting, Alimentary Pharmacology & Therapeutics. (2010) 32, no. 2, 233–243, 10.1111/j.1365-2036.2010.04341.x, 2-s2.0-77953731910, 20456304. 20456304

[22] Wakabayashi S. I. , Kimura T. , Tamaki N. , Iwadare T. , Okumura T. , Kobayashi H. , Yamashita Y. , Tanaka N. , Kurosaki M. , and Umemura T. , AI-Based Platelet-Independent Noninvasive Test for Liver Fibrosis in MASLD Patients, JGH Open. (2025) 9, no. 4, e70150, 10.1002/jgh3.70150, 40191781. 40191781 PMC11969565

[23] Ge J. , Fontil V. , Ackerman S. , Pletcher M. J. , and Lai J. C. , Clinical Decision Support and Electronic Interventions to Improve Care Quality in Chronic Liver Diseases and Cirrhosis, Hepatology. (2025) 81, no. 4, 1353–1364, 10.1097/HEP.0000000000000583, 37611253. 37611253 PMC10998693

