# Supplementary material for: LC‐Pred: A Transformer‐Based Interactive Interface for Liver Cirrhosis Prediction
Source: Int J Hepatol. 2026 May 23;2026:3655128. doi: 10.1155/ijh/3655128 (PMC13198194; doi:10.1155/ijh/3655128)
Supplement: Supplementary file 1 — Supporting Information Additional supporting information can be found online in the Supporting Information section. [file IJH-2026-3655128-s001.docx]

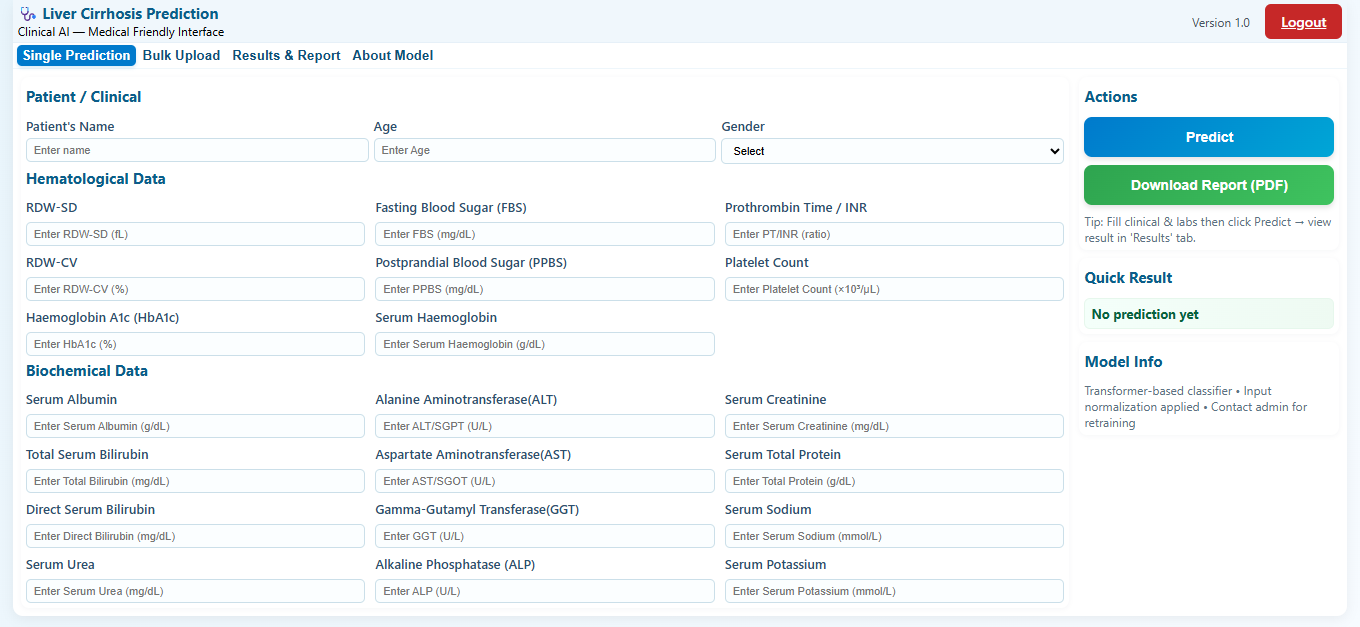


Supplemantary Figure 1: Single Prediction


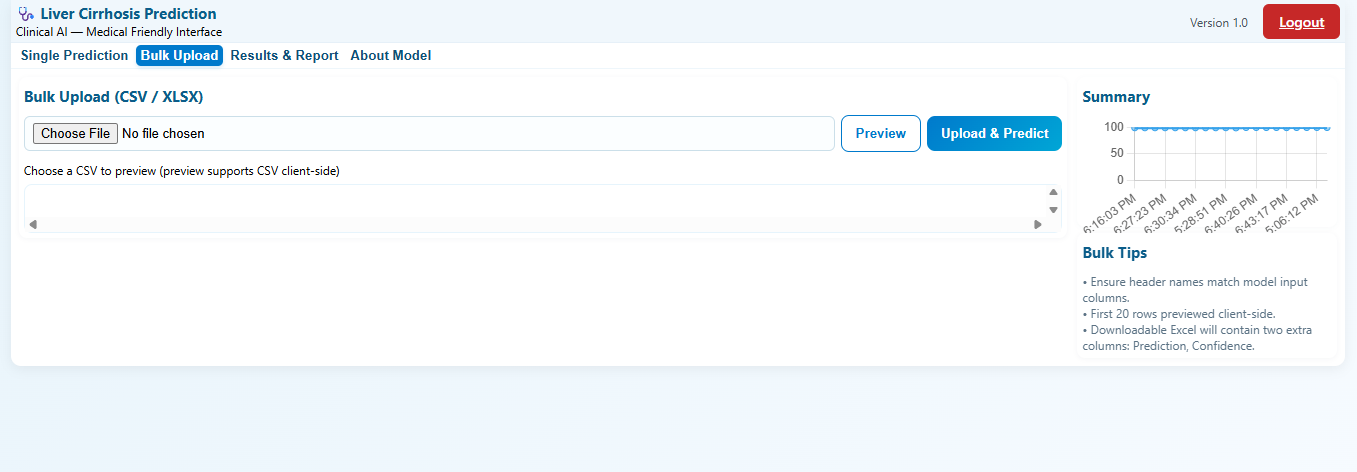


Supplemantry Figure 2: Bulk Upload


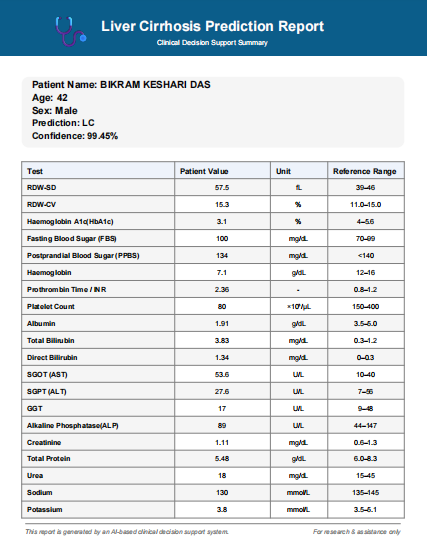


Supplemantry Figure 3: Downloaded Lab Report


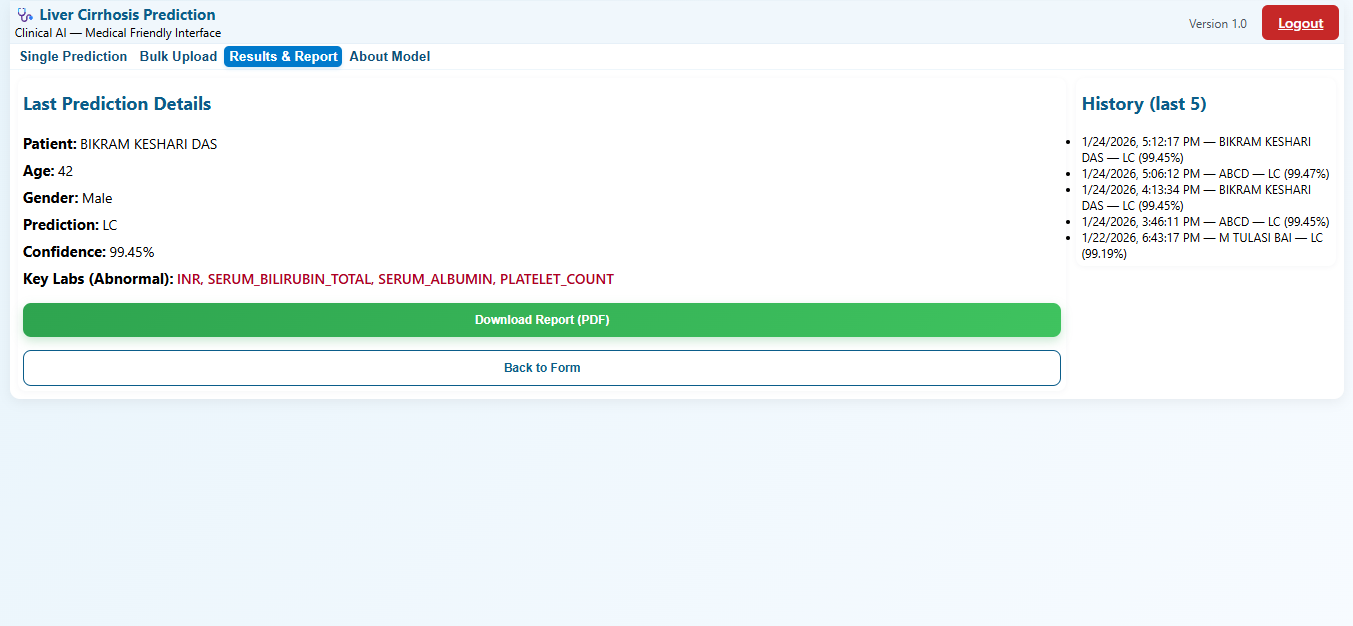


Supplementary Figure 4: Results & Report page (for Single Prediction)


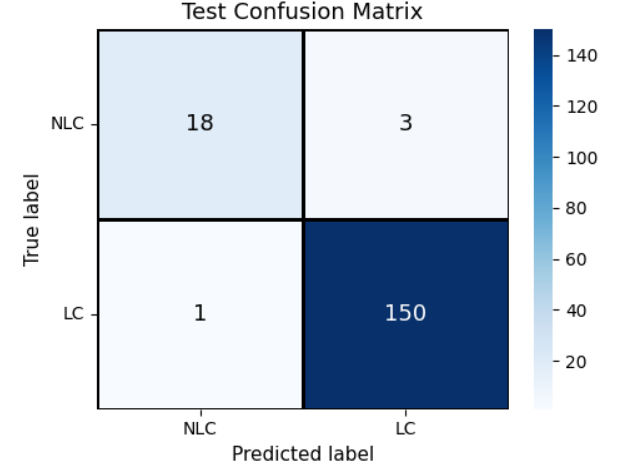


Supplementary Figure 5 : Confusion Matrix of Transformer model
